# Maternal voluntary exercise mitigates oxidative stress and incidence of congenital heart defects in pre‐gestational diabetes

**DOI:** 10.1111/jcmm.14439

**Published:** 2019-06-18

**Authors:** Tana Saiyin, Anish Engineer, Elizabeth R. Greco, Mella Y. Kim, Xiangru Lu, Douglas L. Jones, Qingping Feng

**Affiliations:** ^1^ Department of Physiology and Pharmacology, Schulich School of Medicine and Dentistry University of Western Ontario, Children's Health Research Institute London ON Canada; ^2^ Department of Medicine, Schulich School of Medicine and Dentistry University of Western Ontario, Children's Health Research Institute London ON Canada

**Keywords:** congenital heart defects, exercise, heart development, oxidative stress, pre‐gestational diabetes

## Abstract

Women with pre‐gestational diabetes have a higher risk of producing children with congenital heart defects (CHDs), caused predominantly by hyperglycemia‐induced oxidative stress. In this study, we evaluated if exercise during pregnancy could mitigate oxidative stress and reduce the incidence of CHDs in the offspring of diabetic mice. Female mice were treated with streptozotocin to induce pre‐gestational diabetes, then mated with healthy males to produce offspring. They were also given access to running wheels 1 week before mating and allowed to exercise voluntarily until E18.5. Heart morphology, gene expression, and oxidative stress were assessed in foetal hearts. Maternal voluntary exercise results in a significantly lower incidence of CHDs from 59.5% to 25%. Additionally, diabetes‐induced defects in coronary artery and capillary morphogenesis were also lower with exercise. Myocardial cell proliferation and epithelial‐mesenchymal transition at E12.5 was significantly lower with pre‐gestational diabetes which was mitigated with maternal exercise. Cardiac gene expression of *Notch1*, *Snail1*, *Gata4* and *Cyclin* *D1* was significantly higher in the embryos of diabetic mice that exercised compared to the non‐exercised group. Furthermore, maternal exercise produced lower reactive oxygen species (ROS) and oxidative stress in the foetal heart. In conclusion, maternal exercise mitigates ROS and oxidative damage in the foetal heart, and results in a lower incidence of CHDs in the offspring of pre‐gestational diabetes. Exercise may be an effective intervention to compliment clinical management and further minimize CHD risk in mothers with diabetes.

## 1 INTRODUCTION

Congenital heart defects (CHDs) are structural abnormalities of the heart and coronary arteries that can arise when developmental pathways are disturbed.^1^ An estimated 1%‐5% of live births are affected by CHDs; however, this is believed to be a conservative estimate considering many defects can be asymptomatic at birth and remain undetected until adulthood when severe complications can arise.^2^, ^3^ Studies on CHD aetiology suggest that approximately 15% of cases are attributable to genetic causes, and several environmental factors have been identified.^4^ Of these, maternal pre‐gestational diabetes, either type 1 or type 2 diabetes mellitus diagnosed prior to conception, is a major risk factor with a 4‐5 times increased risk of producing children with CHDs.^5^, ^6^, ^7^ The prevalence of CHDs in both children and adults has increased by 11% and 57%, respectively, from 2000 to 2010.^8^ Some of this increase may be attributable to the rapidly growing prevalence of diabetes in the general population.^9^ This is concerning because CHDs have several associated co‐morbidities including pulmonary hypertension, coronary artery disease, arrhythmia and sudden cardiac death. The total costs of treatment and management is a rising burden on the healthcare system.^3^, ^10^ For these reasons, it is imperative to expand our understanding of the pathogenesis of CHDs and discover viable strategies for prevention.

Maternal physiological adaptations induced by exercise may protect against adverse cardiovascular development in the offspring. Remarkably, a recent study showed that voluntary exercise during pregnancy reduced the maternal age‐related risk of CHDs in offspring with cardiac transcription factor *Nkx2.5* haploinsufficiency.^11^ It is currently unknown how maternal exercise modulates cardiovascular development. This study investigated the effects of maternal voluntary exercise on CHDs induced by pre‐gestational diabetes in mice, and determined possible mechanisms of protection. High‐glucose environments during development disrupt intracellular redox homoeostasis by stimulating reactive oxygen species (ROS) production to exceed levels that can be managed by endogenous antioxidants.^12^, ^13^ Oxidative stress can trigger changes in embryonic gene expression, cause oxidative damage to proteins and disrupt coordinated developmental processes.^14^ Our recent studies showed that inhibition of oxidative stress using an antioxidant, N‐acetylcysteine, resulted in significantly lower incidences of CHDs and coronary artery malformation in the offspring of mice with pre‐gestational diabetes, suggesting an important role of oxidative stress in the pathogenesis of CHDs and coronary artery malformation.^13^, ^15^ Notably, exercise can stimulate the expression and activity of endogenous antioxidants, superoxide dismutase (SOD) and catalase, in aortic and cardiac tissues under diabetes‐induced oxidative stress.^16^ However, there is limited knowledge on whether maternal exercise can affect foetal oxidative stress in utero. Furthermore, it is not known if maternal exercise protects against CHDs induced by pre‐gestational diabetes. We hypothesized that exercise would mitigate oxidative stress and produces lower incidences of CHDs and coronary artery malformation in the offspring of diabetic mothers. We tested this hypothesis using streptozotocin (STZ)‐induced pre‐gestational diabetes and voluntary wheel running in mice.

## 2 MATERIALS AND METHODS

### 2.1 Animals

This study was performed in accordance with the guidelines of the Canadian Council on Animal Care and approved by the Animal Care Committee at Western University, Canada (Protocol #2016‐099). All efforts were made to minimize animal suffering and minimize the number of animals used.

Female C57BL/6 mice were purchased from Jackson Laboratory (Bar Harbor, Maine). Animals had access to standard chow and water ad libitum, and were housed in 12 hours light/dark cycle. At 8 weeks old, mice were given intraperitoneal injections of STZ (Sigma) at a dose of 50 mg/kg body weight/day for five consecutive days.^17^ Control mice received saline injections. One week post‐injection, non‐fasting blood glucose was measured using the One Touch Ultra2 glucose meter (LifeScan) to determine diabetic status. Mice with blood glucose levels exceeding 11 mmol/L were considered diabetic. Diabetic and control female mice were then placed individually in a cage with or without a free‐spinning running wheel (Mouse running wheel, 5.1 × 10.2 cm, Columbus Instruments, Columbus, OH) for one week to allow acclimatization. The females were then bred with healthy 10‐12 weeks old WT males overnight in cages with no running wheel, and returned to their original cage in the morning. The presence of a vaginal plug indicated embryonic day (E) 0.5. The number of wheel turns per day was recorded during the entire pregnancy using the Multi‐Device Channel Interface software (Columbus Instruments, Columbus, OH). The distance run per day was calculated by multiplying the number of running wheel rotations in 24 hours by the circumference of the wheel. Non‐fasting blood glucose measurements were taken at the same time every morning to monitor glycemic levels throughout pregnancy from E0.5 to E18.5. There were four groups of offspring: control, exercise control, as well as offspring of maternal diabetes (OMD) with and without maternal exercise.

### 2.2 Histological analysis of CHD

Foetal samples were collected on E18.5, then fixed in 4% paraformaldehyde for 18 hours at 4°C. Samples were then dehydrated in ethanol and embedded in paraffin to be sectioned into 5 µm thick serial sections using a Leica RM2255 microtome. Transverse sections began at the level of the thymus (above the aortic arch) and continued until the apex of the heart. To diagnose CHDs, sections were stained in hematoxylin and eosin (H/E), and examined under a light microscope (Observer D1, Zeiss).^13^, ^17^

### 2.3 Immunohistochemistry

Foetal heart sections were subjected to antigen retrieval in citric acid buffer (0.01 M, pH 6.0) for 12 minutes at 94°C in a microwave (BP‐111, Microwave Research & Applications). E18.5 heart sections were then stained using anti‐α‐smooth muscle actin primary antibody (1:3000; Sigma), biotinylated griffonia simplicifolia lectin‐1 (1:200; Vector Laboratories) and anti‐sex‐determining region Y protein antibody (1:200, Santa Cruz) to visualize coronary arteries, capillaries and Y chromosome, respectively. To analyse proliferation and epicardial epithelial‐to‐mesenchymal transition (EMT), E12.5 heart sections were incubated in anti‐phosphorylated histone H3 (pHH3, 1:500; Abcam) and anti‐Wilm's tumor‐1 (Wt1, 1:300; Calbiochem) primary antibodies, respectively. Primary antibodies were left on overnight at room temperature in humidity chambers. Sections were subsequently incubated for one hour at room temperature with secondary antibodies; biotinylated donkey anti‐goat IgG (1:500), biotinylated goat anti‐rabbit IgG (1:500) or biotinylated goat anti‐mouse IgG (1:500) (Vector Laboratories). Slides were imaged using the Zeiss Observer D1 microscope and AxioVision Rel 4.7 Software. All quantifications of histological images were performed using ZEN microscope software (Zeiss).^13^, ^15^, ^17^

### 2.4 AMIRA 3D reconstruction

AMIRA® software (Template Graphics Software) was used to reconstruct E18.5 sections of the heart into a three‐dimensional (3D) model. Sections stained with α‐smooth muscle actin were used so that coronary vasculature could be visualized and reconstructed. Images were taken at each 25‐µm section of the heart. The compact myocardium and coronary arteries were labelled manually in each section. AMIRA calculated the volume of the labelled components and these values were used to calculate the ratio of coronary artery volume to myocardial volume.^15^, ^18^

### 2.5 Real‐time quantitative RT‐qPCR analysis

Embryonic E12.5 ventricles were isolated, washed in ice cold phosphate buffered saline and then snap frozen in liquid nitrogen. Total RNA was extracted from E12.5 embryonic ventricles using the RNeasy Mini Kit (Qiagen) with the manufacturer's instructions and reagents. Reverse transcription with M‐MLV reverse transcriptase (Invitrogen) was performed using 0.5 µg of total RNA. Following reverse transcription, real‐time quantitative PCR amplification was performed using EvaGreen qPCR MasterMix (Applied Biological Materials). Primers were designed to amplify the genes *Nkx2.5*, *Gata4*, *Bmp10*, *Cyclin* *D1*, *Hif‐1α*, *VEGFα*, *Notch1*, *Snail1* and *28S* (Table 1). Eppendorf MasterCycler Realplex (Eppendorf) was used to amplify qPCR mixtures for 35 cycles at temperatures set in accordance with the primer melting temperatures. The Ct values of target genes were normalized to 28S ribosomal RNA.^15^, ^17^

**Table 1:** Primer sequences and accession numbers for primers used in RT‐qPCR

Gene	Accession no.	Product size	Primer sequence (5'→3')
Nkx2.5	NM_008700.2	162	F: GACAGCGGCAGGACCAGACT R: CGTTGTAGCCATAGGCATTG
Gata4	NM_008092.4	137	F: CACTATGGGCACAGCAGCTC R: GCCTGCGATGTCTGAGTGAC
Bmp10	NM_009756.3	239	F: CCACTCGGATCAGGAGGAAC R: CACACAGCAGGCTTTGGAAG
eNOS	NM_008713.4	166	F: ATTTCCTGTCCCCTGCCTTCC R: ATGGTTGCCTTCACACGCTTC
Cyclin D1	NM_007631.2	155	F: CTGACACCAATCTCCTCAACG R: CTCACAGACCTCCAGCATCCA
Hif1‐α	NM_001313919	237	F: CAGCCTCACCAGACAGAGCA R: GTGCACAGTCACCTGGTTGC
Notch1	NM_008714.3	141	F: CAGCTTGCACAACCAGACAGA R: TAACGGAGTACGGCCCATGT
Snail1	NM_011427.3	133	F: CACACGCTGCCTTGTGTCT R: GGTCAGCAAAAGCACGGTT
28S	NR_003279.1	178	F: GGGCCACTTTTGGTAAGCAG R: TTGATTCGGCAGGTGAGTTG

Abbreviations: F: Forward, R: Reverse.

### 2.6 Western blot analysis

Snap frozen E14.5 ventricles were used to measure levels of phosphorylated eNOS (p‐eNOS) protein by Western blotting. Protein was isolated in PhosphoSafe buffer (Fisher Scientific) and lysed by sonication. Forty‐five micrograms of total protein per well were separated by 8% SDS‐PAGE, then transferred onto PVDF membrane. The membrane was blocked for 1 hour in TBST with 5% skim milk, then bands were tagged using anti‐phospho‐NOS3 primary antibody (1:1000; Santa Cruz). Additionally, membranes were also blotted for total eNOS using anti‐NOS3 primary antibody (1:1000; Santa Cruz), and anti‐α‐actinin antibody (1:1000; Sigma) which served as the loading control. Horse radish peroxidase‐conjugated secondary antibodies (1:2500; Bio‐Rad) were used to detect protein bands using the enhanced chemiluminescence method. Signal intensity was quantified by densitometry using the AlphaEaseFC image analysis software.^17^, ^18^

### 2.7 Analysis of Superoxide and Lipid peroxidation

Frozen embryonic E14.5 ventricles from each group were embedded in Tissue‐Tek® OCT Compound (Sakura Finetek) and sectioned into 8‐µm sections using a cryostat (CM1950, Leica, Wetzlar, Germany). Dihydroethidine (DHE, Invitrogen Life Technologies), the molecular probe for superoxide, was used to measure relative ROS levels by quantifying fluorescence densitometry. Sections were incubated in 2 µmol/L of DHE for 30 minutes at 37**°**C in a light‐protected humidity chamber. Fluorescence was imaged using the microscope (Observer D1, Zeiss). Three to five images were taken from three different sections per heart sample at a fixed exposure time. AxioVision software was used to quantify the signal intensity per area of myocardium. To assess cellular oxidative stress, lipid peroxidation was measured in E14.5 frozen heart sections. Slides were incubated with anti‐4‐hydroxynonenal primary antibody (1:1000; Applied Biological Materials) overnight at room temperature. A fluorescent‐labelled secondary antibody (LI‐COR Biosciences) was used to visualize lipid peroxidation, followed by Hoechst stain to label the nuclei. Densitometry quantification of the fluorescence signal was performed the same way as DHE quantification.^15^, ^17^

### 2.8 Statistical analysis

Data are presented as means ± SEM. Statistical analysis was performed using two‐way ANOVA followed by Tukey's multiple comparisons test (GraphPad Prism, version 6.0). A chi‐squared test was used to analyse the incidence of CHD. A *P* < 0.05 was considered statistically significant.

## 3 RESULTS

### 3.1 Exercise and blood glucose levels monitored during gestation

**Figure 1:**
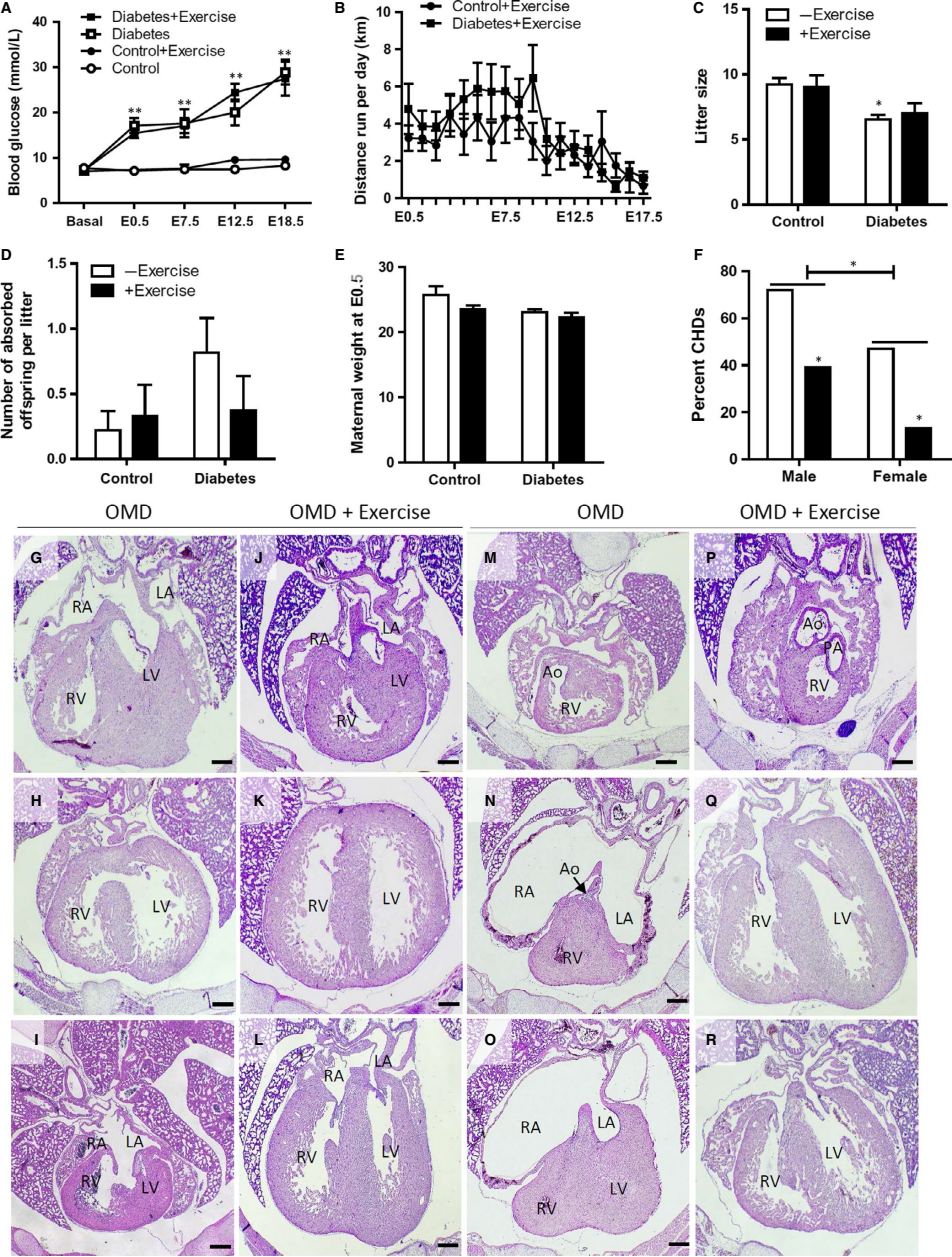
Effects of maternal exercise on blood glucose, litter size and congenital heart defects in the offspring of maternal diabetes (OMD). (A), Non‐fasting blood glucose levels during gestation in streptozotocin **(**STZ)‐treated and control female mice with and without exercise (n = 3‐5). Basal indicates before STZ injection. (B), Running distance per day of gestation from E0.5‐17.5 (n = 4‐7 per group). (C), Offspring litter size. (D), Average number of absorbed offspring per litter. (E), Maternal body weight at E0.5 (n = 7‐11 per group). (F), Per cent congenital heart defects in males and females of OMD. Data are means ± SEM. *P < 0.05, **P < 0.01 vs control. Without maternal exercise, E18.5 hearts of OMD show ASD (G), VSD (H), AVSD (I), DORV (M), HLHS and HRHS (N and O) with ASD, aortic stenosis and hypoplasia of LV, RV, mitral and tricuspid valves. Corresponding E18.5 normal hearts of OMD with maternal exercise are shown in J‐L and P‐R RA: right atrium, LA: left atrium, Ao: aorta, PA: pulmonary artery. Scale bars are 200 µm

Prior to STZ injection, there was no difference in blood glucose levels between the four groups (Figure 1A). For both exercised and non‐exercised diabetic mice, non‐fasting blood glucose levels were significantly higher at E0.5 compared to controls (*P* < 0.05). As pregnancy progressed towards E18.5, blood glucose levels gradually increased for the diabetic groups. There was no significant difference in the hyperglycemic state of mice that exercised compared to mice that did not exercise at any time point in gestation. Diabetic mice ran approximately 2‐5 km per day in the first 9 days of gestation, after which the levels of exercise gradually declined as the dams grew larger (Figure 1B). Mean litter size was significantly smaller for diabetic dams with no exercise, compared to control (Figure 1C, *P* < 0.05). Furthermore, litters from diabetic dams without exercise had a trend of higher numbers of absorbed offspring (Figure 1D). There was no significant difference in maternal body weight at E0.5 among the four groups (Figure 1E).

### 3.2 Incidence of CHDs was lower in offspring of exercised mice with pre‐gestational diabetes

The incidence of CHDs in E18.5 OMD without exercise was 59.5% (Table 2). Male OMD had a significantly higher incidence of CHDs (72.2%) than females (47.4%), and maternal exercise resulted in lower incidences of CHDs to 29.4% and 13.3% for male and female OMD, respectively (*P* < 0.05, Figure 1F). The commonly diagnosed defects were ASD (43.2%), VSD (10.8%), atrioventricular septal defect (AVSD), double outlet right ventricle (DORV) and stenosis of pulmonary and aortic valves (Table 2, Figure 1G‐M). Overall, the offspring of diabetic mice that exercised had a significantly lower incidence of CHDs (25%). The incidence of ASD was also lower (15.6%), while VSD, DORV or AVSD was not present in the exercised group (Table 2). In the OMD group, one foetus had a hypoplastic left heart syndrome (HLHS) and a hypoplastic right heart syndrome (HRHS), which feature hypoplasia of LV, RV, aorta and pulmonary artery with an ASD, patent ductus arteriosus and atresia of mitral and tricuspid valves (Figure 1N,O). Interestingly, OMD with exercise also had an HLHS but without HRHS (Table 2).

**Table 2:** Incidence of congenital heart defects in the offspring of diabetic and control female mice with and without voluntary exercise

	WT Control (n = 20/3 Litters)		OMD (n = 37/7 Litters)		Exercise control (n = 20/3 Litters)		OMD + Exercise (n = 32/7 Litters)	
	n	%	n	%	n	%	n	%
Normal	20	100	16	**43.2**	20	100	24	**75**
Abnormal	0	0	21	**56.8** **	0	0	8	**25** ^†^
ASD	0	0	16	43.2**	0	0	5	15.6^†^
VSD	0	0	4	10.8	0	0	0	0
AVSD	0	0	2	5.4	0	0	0	0
DORV	0	0	2	5.4	0	0	0	0
PVS	0	0	10	27*	0	0	4	12.5
AVS	0	0	7	18.9*	0	0	2	6.2
HLHS	0	0	1	2.7	0	0	1	3.1
HRHS	0	0	1	2.7	0	0	0	0

Note

Data was analysed using the chi‐squared test.

Abbreviation: ASD, atrial septal defect; AVS, aortic valve stenosis; AVSD, atrioventricular septal defect; DORV, double outlet right ventricle; HLHS, hypoplastic left heart syndrome, which includes a hypoplastic LV, aortic stenosis, patent ductus arteriosus (PDA) and ASD; HRHS, hypoplastic right heart syndrome, which includes a hypoplastic RV, pulmonary stenosis, PDA and ASD; OMD, offspring of maternal diabetes; PVS, pulmonary valve stenosis; VSD, ventricular septal defect.

* *P* < 0.05,

** *P* < 0.01 vs control,

^†^ *P* < 0.05 vs OMD.

**Figure 2:**
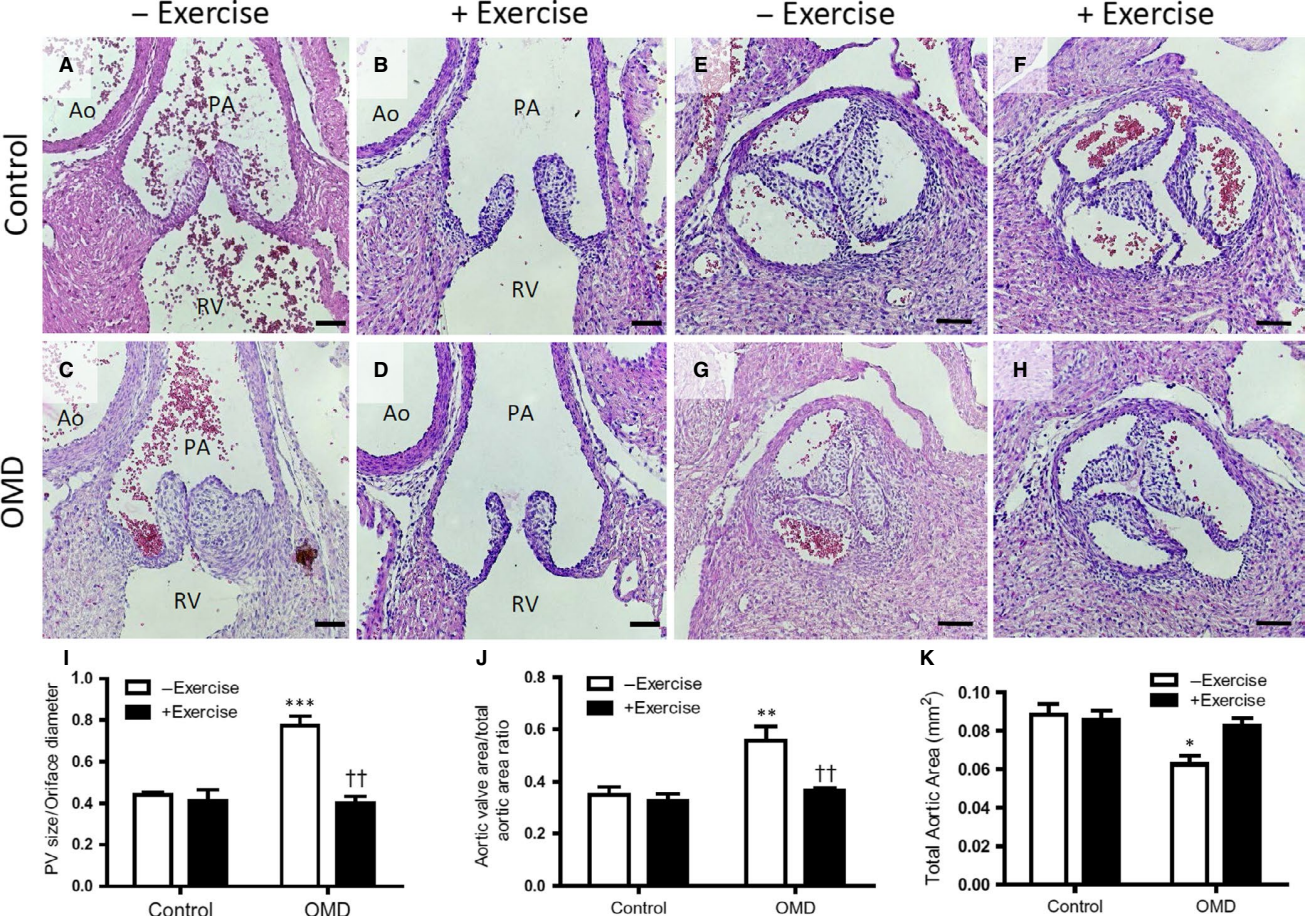
Maternal exercise prevents pulmonary and aortic valve defects in offspring of maternal diabetes (OMD). Representative images of pulmonary and aortic valves from E18.5 hearts of normal controls (A, B, E, F) and OMD (C, D, G, H) with and without maternal exercise. Abnormalities of pulmonary (C) and aortic (G) valves in offspring of diabetic dams are prevented with maternal exercise (D, H). (I), Pulmonary valve size to orifice diameter ratio. (J), Ratio of aortic valve area to total aortic area. (K), Total aortic area. *P < 0.05, **P < 0.01 vs control. ^††^ *P* < 0.01 vs OMD without exercise. Data are means ± SEM. n = 6‐7 hearts per group. NCC, non‐coronary cusp; RCC, right coronary cusp; LCC, left coronary cusp. Scale bars are 50 µm

Offspring from diabetic dams with exercise had lower incidences of pulmonary (12.5%) and aortic valve stenosis (6.2%) compared to OMD without exercise (27% and 18.9%, respectively). Representative images of pulmonary valves (Figure 2A‐D) and aortic valves (Figure 2E‐H) at E18.5 show poorly re‐modelled valves in the OMD group without exercise. Offspring from this group had significantly thicker pulmonary and aortic valves, and smaller aortic luminal diameters compared to those of controls, which was prevented with maternal exercise (2I‐K).

### 3.3 Effect of maternal exercise on coronary artery and capillary malformations in the offspring of diabetic mice

**Figure 3:**
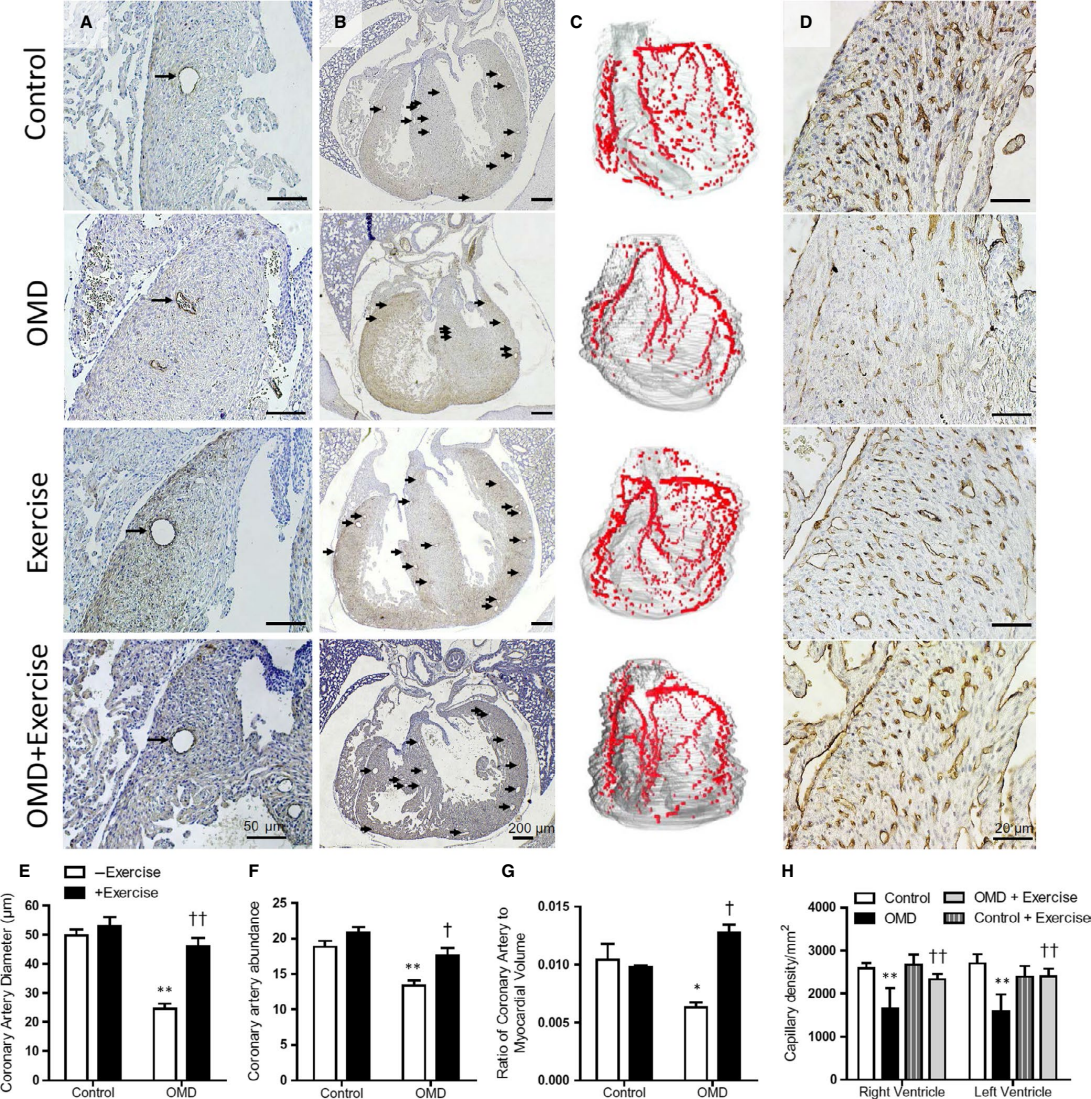
Maternal exercise prevents coronary artery and capillary abnormalities in offspring of maternal diabetes (OMD). Representative images of coronary artery size (A) and abundance (B) in E18.5 hearts stained with anti‐α‐smooth muscle actin antibody. (C), Three‐dimensional reconstruction of coronary arteries. (D), Lectin‐1 staining shows capillaries (brown colour) in the ventricular myocardium of E18.5 hearts. Quantification coronary artery diameter (E) and abundance (F) in E18.5 hearts of control and OMD with and without maternal exercise (n = 4‐7 hearts per group). (G), AMIRA software was used to quantify coronary artery and myocardial volume of the heart using 5 µm sections 25 µm apart, from the level of the pulmonary valves to the apex of the heart (n = 3 per group). (H), Capillary density is normalized to the area of RV and LV myocardium of OMD with and without exercise (n = 4‐6 per group). Data are means ± SEM. *P < 0.05, **P < 0.01 vs control. ^†^ *P* < 0.05, ^††^ *P* < 0.01 vs OMD without exercise

Coronary artery malformation was evaluated in E18.5 foetal hearts using immunostaining with α‐smooth muscle actin antibody, which labelled smooth muscle cells of coronary arteries (representative images Figure 3A,B). The coronary artery diameter was determined in the ventricular myocardium at the four chamber view by measuring the widest luminal diameter in three sections. Hearts collected from diabetic mice that did not exercise had significantly smaller coronary artery diameter (Figure 3E). The relative coronary artery abundance was quantified by averaging the number of positively stained ventricular arteries per section in three sections of the heart. The OMD group had significantly fewer coronary arteries per section compared to both controls (Figure 3F). Three‐dimensional reconstruction of the coronary vasculature and myocardium also showed less branching, as well as narrower and shorter coronary arteries in the OMD group compared to other groups (Figure 3C). The ratio of the coronary artery volume to the myocardial volume was significantly smaller in this group (Figure 3G). These abnormalities of coronary artery development in foetal hearts from diabetic mothers were prevented with maternal exercise. Finally, capillaries visualized by immunostaining with lectin‐1 showed evidence of impaired capillary development in the offspring of diabetic mice (Figure 3D). Capillary abundance was quantified in three sections at the widest four chamber view of the heart. In both the right and left ventricular myocardium, the number of capillaries normalized to the area of myocardium was significantly lower in the OMD group compared to respective controls, and this defect was prevented with maternal exercise (Figure 3H).

### 3.4 Effects of maternal exercise on cell proliferation and epicardial EMT in the developing embryonic heart

**Figure 4:**
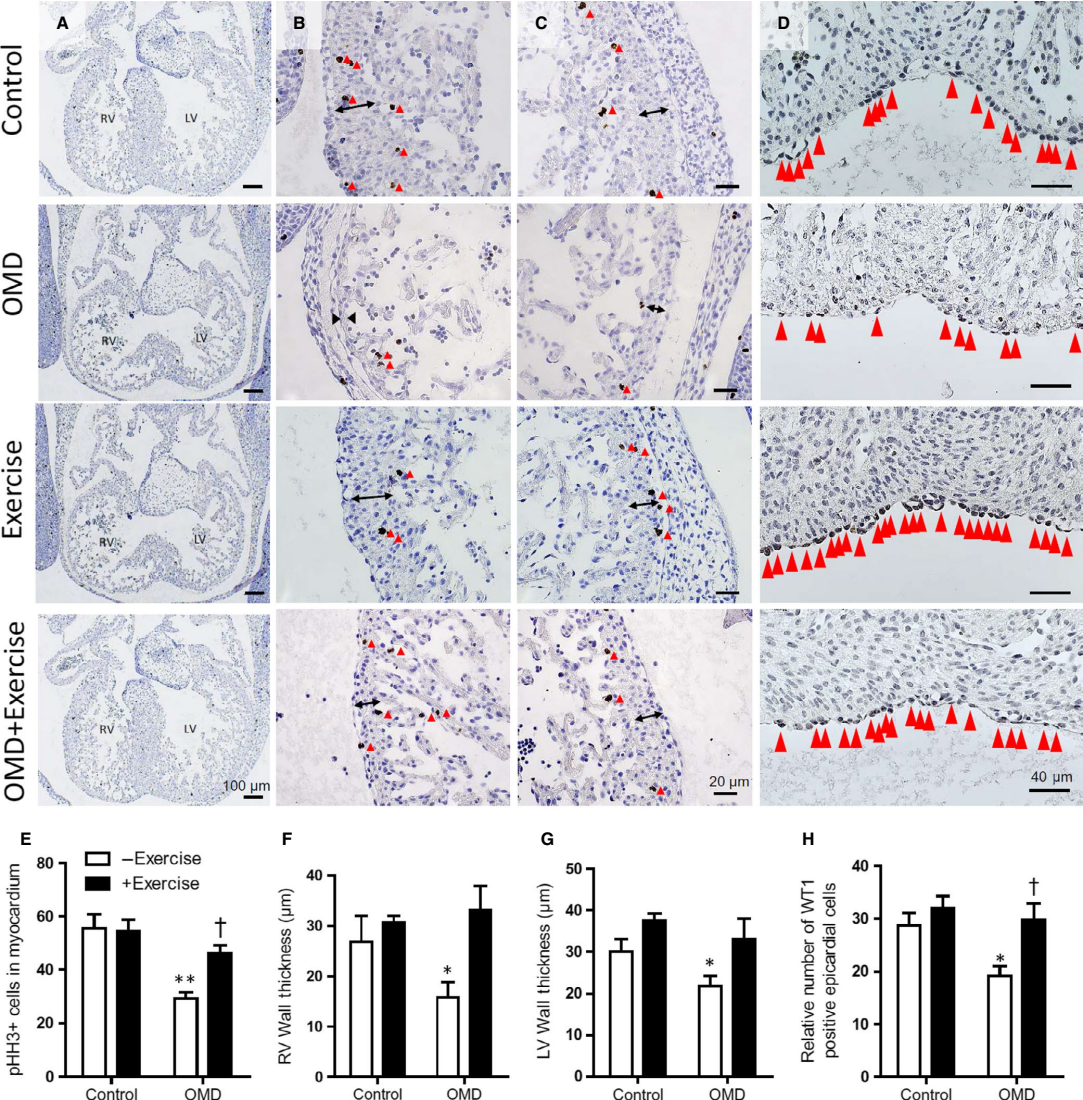
Maternal exercise improves cell proliferation and epithelial‐to‐mesenchymal transition in E12.5 hearts of offspring of maternal diabetes (OMD). (A‐C), Sections of E12.5 hearts immunostained with anti‐phosphorylated histone H3 (pHH3) antibody‐marking proliferating cells. (B, C), Representative images of ventricular wall thickness in E12.5 hearts. Red arrow heads indicate pHH3‐positive cells (dark brown). (D), E12.5 epicardium immunostained with anti‐WT1 antibody. Red arrow heads indicate WT1‐positive cells. E, Number of pHH3‐positive cells, (F), RV wall thickness and (G), LV wall thickness (n = 3‐6 per group). (H), Quantification of the number of WT1‐positive cells normalized to epicardial length (n = 4‐5 per group). *P < 0.05 vs control. ^†^ *P* < 0.05 vs OMD without exercise. Data are means ± SEM

Cell proliferation and epicardial EMT are required for proper morphogenesis of the heart and coronary vasculature.^19^, ^20^ Proliferating cells were marked by immunostaining E12.5 hearts with pHH3 antibody (Figure 4A‐C), as histone H3 is specifically phosphorylated during mitosis.^21^ Likewise, cells undergoing epicardial EMT were labelled by immunostaining with anti‐Wt1 antibody (Figure 4D). Embryonic hearts from diabetic mice were smaller overall and appeared less developed compared to other groups. The number of pHH3 positive cells were counted in three heart sections at the level of the AV cushions. Notably, the average number of pHH3 positive cells in the ventricular myocardium and AV endocardial cushion were significantly lower in the OMD group without exercise, compared to other groups (Figure 4E). Additionally, the thickness of the left and right ventricular walls was also significantly thinner for this group at E12.5 (Figure 4F,G). On the other hand, the ventricular wall thickness of embryonic hearts from the OMD + exercise group was not significantly different from controls. Embryonic Wt1 expression was quantified in the epicardium lining the ventricles, at E12.5. Wt1 positive cells were counted in three sections at the level where all four chambers of the heart were visible and normalized to the length of epicardium. Embryos from dams with maternal diabetes had a significantly lower number of Wt1‐positive epicardial cells compared to those of controls (Figure 4H). This difference was eliminated in embryos from diabetic mice with voluntary exercise.

### 3.5 Effects of maternal exercise on gene expression and oxidative stress in embryonic hearts

**Figure 5:**
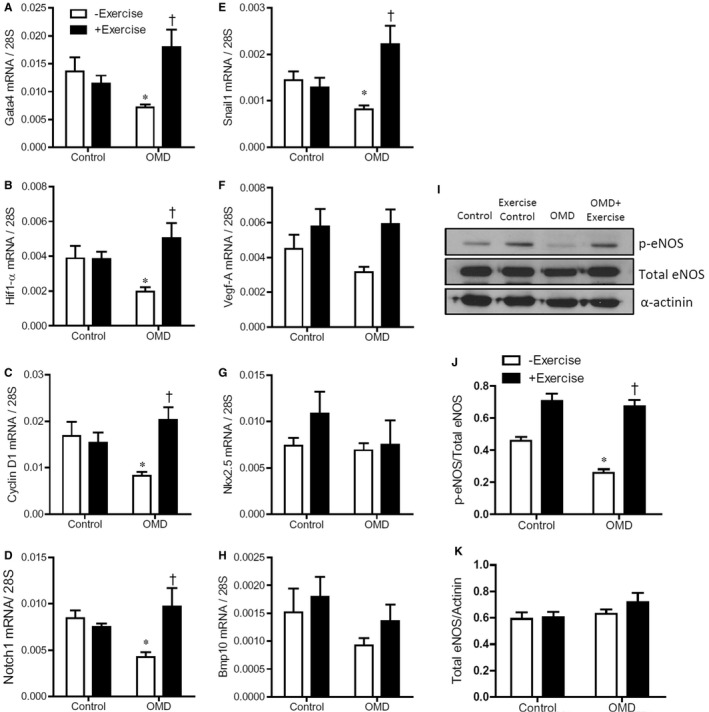
Maternal exercise restores gene expression and eNOS phosphorylation in E12.5 hearts of offspring of maternal diabetes (OMD). (A‐H), Cardiac mRNA levels of *Gata4*, *Hif‐1α*, *Cyclin D1*, *Notch1*, *Snail1*, *Vegf‐A*, *Nkx*2.5 and *Bmp10* in OMD with and without maternal exercise (n = 5‐8 hearts per group). (I), Representative Western blot depicting p‐eNOS and total eNOS at 130 kDA and α‐actinin at 100 kDa. (J), Densitometry quantification of the ratio of p‐eNOS to total eNOS (n = 3 hearts per group) and (K), ratio of total eNOS protein to α‐actinin quantified by densitometry (n = 3 hearts per group). *P < 0.05 vs control without exercise. ^†^ *P* < 0.05 vs OMD without exercise. Data are means ± SEM

To investigate molecular pathways regulating cardiogenesis and vasculogenesis during embryonic development, real‐time RT‐PCR was used to measure relative gene expression levels of *Gata4,* a critical factor for heart development, and *Hif‐1α*, involved in vasculogenesis. Additionally, *CyclinD1* gene expression was also measured as a marker for cell proliferation and *Notch1* and *Snail1* were measured as markers of EMT. The expression levels of *Gata4*, *Hif1a*, *Cyclin* *D1*, *Notch1* and *Snail1* were lower in E12.5 embryonic hearts from diabetic mice compared to controls and were restored or up‐regulated by maternal exercise (Figure 5A‐E). There was no significant difference in the mRNA levels of *VEGF‐a*, *Nkx*2.5 and *Bmp10* (Figure 5F‐H). To evaluate whether maternal exercise also had an impact on foetal enzyme function, eNOS phosphorylation was measured using Western blotting (Figure 5I). The ratio of p‐eNOS to total eNOS was significantly lower in the OMD embryos compared to other groups. Moreover, the ratio was significantly higher in both exercise groups compared to the control group (Figure 5J). There was no significant difference in relative levels of total eNOS (Figure 5K).

**Figure 6:**
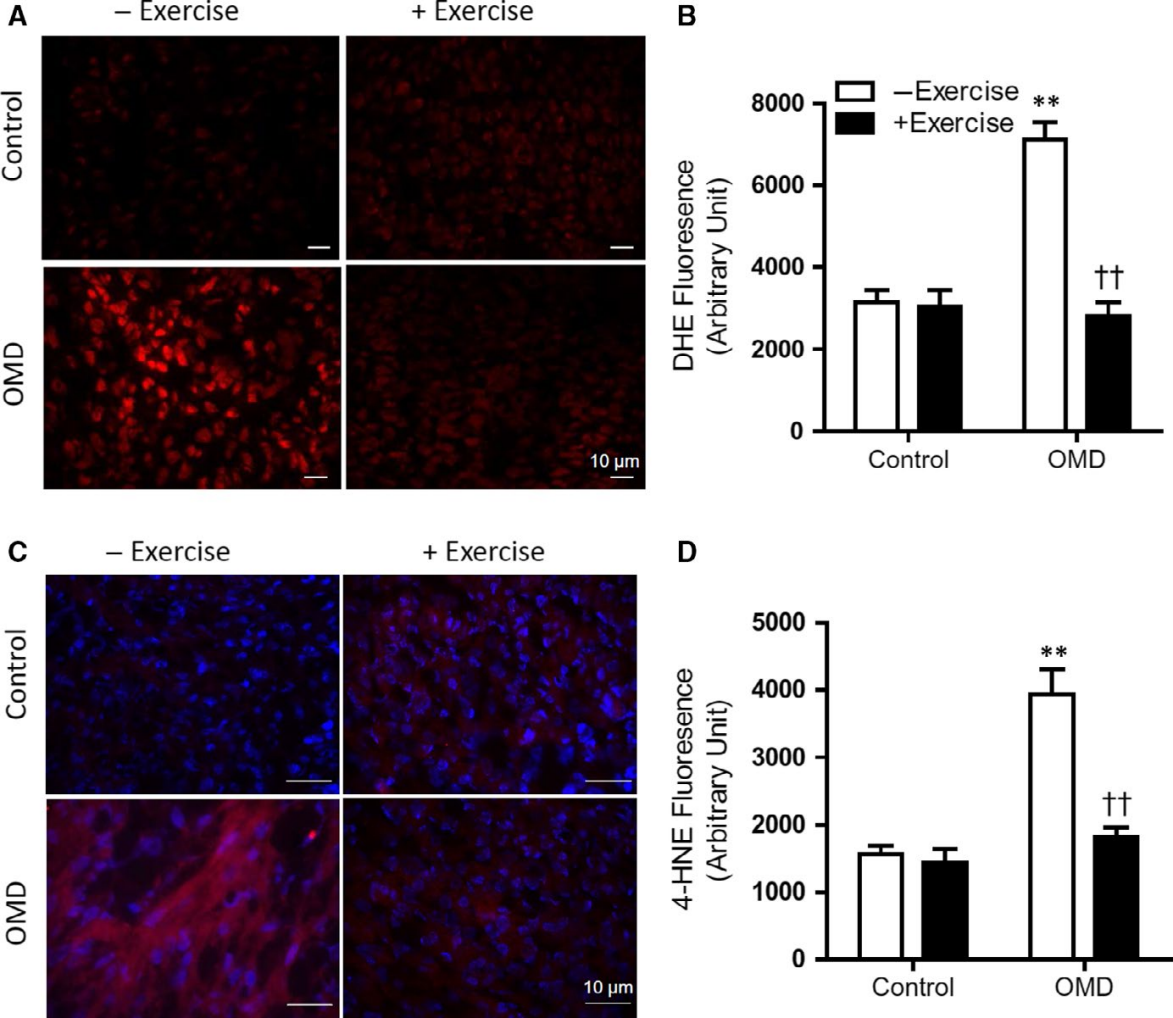
Maternal exercise reduces oxidative stress in hearts of offspring of maternal diabetes (OMD). (A), Representative images of E14.5 hearts stained with dihydroethidine (DHE) probe for superoxide content imaged at 20‐ms exposure. (B), Quantification of relative fluorescence by densitometry normalized to area (n = 5 hearts per group). (C), Representative images of E14.5 embryonic hearts immunostained with anti‐4‐HNE to measure lipid peroxidation. Images were taken at 100‐ms exposure. (D), Quantification of relative fluorescence by densitometry normalized to area (n = 3‐4 hearts per group). **P < 0.01 vs control. ^††^ *P* < 0.01 vs OMD without exercise. Data are means ± SEM

Lastly, to determine the effects of maternal exercise on redox homoeostasis in heart development, markers for oxidative damage were evaluated at E14.5. DHE probe was used to measure superoxide levels, and immunostaining with anti 4‐hydroxynonenal (4‐HNE) antibody was performed to visualize lipid peroxidation (Figure 6A,C). Images were taken at a constant exposure in three sections and the fluorescence intensity was quantified by densitometry. There were significantly higher levels of oxidative stress markers (superoxide and 4‐HNE) in the OMD group without exercise, which was normalized in the OMD group with maternal exercise (Figure 6B,D).

## 4 DISCUSSION

Finding effective strategies to modify disease risk is the focus of preventative healthcare, which has become increasingly relevant as the prevalence of chronic disease continues to rise. Risk factors such as pre‐gestational diabetes have the potential to be controlled through pre‐natal interventions. The aim of this study was to evaluate maternal exercise as an intervention to counteract the damaging effects of pre‐gestational diabetes on heart development, as well as to highlight potential pathways that might lead to these benefits. The results of this study demonstrate that maternal exercise effectively produced a lower incidence of CHDs caused by pre‐gestational diabetes in a mouse model from 59.5% to 25%, while also mitigating coronary artery and capillary malformations in the offspring of diabetic mice. Our data agree with the findings of Schulkey et al,^11^ which was the first study to show that the risk of CHD could be modified by maternal exercise in Nkx2.5^+/−^ offspring. However, the mechanism by which exercise benefits heart development was not identified, and it was unknown if maternal exercise could benefit CHDs induced by non‐genetic factors such as hyperglycemia. For the first time, we demonstrate that pre‐natal maternal exercise results in a lower incidence of CHDs, and has significant effects on embryonic gene expression and redox balance in the hearts of offspring affected by pre‐gestational diabetes.

Streptozotocin has been used in past studies to induce congenital malformations in animal models of type 1 diabetes.^13^, ^15^, ^22^ To avoid unintended teratogenic effects, STZ was administered 1 week prior to mating. The drug is rapidly metabolized and has a plasma half‐life of 5 minutes in mice, so it is expected to have been completely eliminated from the maternal environment by the time mating occurred.^23^ No significant difference was found in running distance between control and diabetic mice. There was also no significant difference in the non‐fasting blood glucose levels of both diabetic groups regardless of exercise, indicating that results of this study are independent of hyperglycemic status. In this study, maternal exercise was effective in limiting the severity of defects induced by pre‐gestational diabetes. According to the categorization used by Hoffman et al,^24^ defects range from mild to complex based on the need for interventional expertise to manage the CHD. Generally speaking, defects such as AVSD, DORV and HLHS are more severe and complex than VSD, ASD and valve stenosis. Our model of untreated pre‐gestational diabetes produced a range of CHDs, which agreed with our previously studies using the same model.^13^, ^17^ A higher number of complex defects were found in the OMD group without exercise (AVSD n = 2, DORV n = 2 and HLHS with HRHS n = 1) compared to OMD with maternal exercise (HLHS n = 1). The incidence of malformations of the coronary vasculature and capillaries were markedly lower compared to the non‐exercised group. All observed malformations have also been linked to pre‐gestational diabetes in humans.^25^, ^26^

A defect in the morphology of the heart and its vasculature indicates a misstep in one or more developmental processes. Our data provide evidence that exercise protects against impaired proliferation and epicardial EMT during embryonic development. Abnormal cell proliferation is evident in E12.5 hearts in the OMD group, which were visibly smaller, with significantly thinner ventricular myocardium compared to other groups. The fewer number of pHH3 immunostained cells in E12.5 OMD hearts indicate insufficient proliferation compared to control levels. This was further supported by gene expression analysis, which showed that embryonic hearts in the offspring of diabetic mice had significantly lower expression of *Cyclin* *D1*, a marker of cell proliferation, as well as cardiogenic transcription factor *Gata4*, an essential regulator of heart development that also plays a role in potentiating proliferation during cardiac septation.^27^ On the other hand, the mRNA level of *Hif‐1α,* normally involved in promoting proliferation, EMT, and migration during vascular development,^28^, ^29^ was significantly lower in the non‐exercised offspring of diabetic dams compared to those with exercise. Other important factors for EMT include *Notch1* and *Snail1*, both of which had significantly lower mRNA levels in the OMD group. *Notch1* expression is required for vascular development, valve primordial formation and outflow tract development.^30^, ^31^ Histological analysis of epicardial Wt1 expression at E12.5 revealed fewer cells expressing this critical transcription factor, which is necessary for the repression of the epithelial phenotype of the epicardium during EMT at this time point.^32^ In other studies, *Wt1*‐null mice have been shown to have diminished cell proliferation in the compact myocardium and inadequate coronary plexus formation.^33^ As such, loss of *Wt1* and downstream *Snail1* expression at E12.5 likely contributed to abnormal coronary artery and capillary morphology observed in E18.5 foetal hearts from the OMD group. Our results indicate that several important regulators of cardiovascular development are altered by maternal diabetes, with the potential to cause the spectrum of CHDs. Maternal exercise was protective against these changes during embryonic development, reducing the pathogenesis of CHDs and vascular malformations.

Studies have shown that oxidative stress is a major driver of CHD pathogenesis.^13^, ^15^, ^34^, ^35^ In our study, E14.5 embryonic hearts from the offspring of diabetic mice had significantly higher levels of superoxide and lipid peroxidation, an indicator of oxidative damage.^36^ Conversely, with maternal exercise, levels were normalized to control levels despite the diabetic dams having comparable hyperglycemic status. These results demonstrate that maternal exercise may improve the ability to maintain redox balance in in the foetal heart, preventing hyperglycemia‐induced oxidative damage. In adults, exercise is known to transiently elevate ROS production, but paradoxically lead to improved redox homoeostasis capacity by up‐regulating antioxidant defences.^37^, ^38^, ^39^ A recent study by Chung et al.^40^ found that maternal exercise during pregnancy lead to increased mitochondrial enzymatic activity and ATP production, while decreasing hydrogen peroxide levels in foetal mouse hearts. However, whether maternal exercise may lead to up‐regulation of endogenous antioxidants and/or, reduced ROS production due to improved mitochondrial efficiency in our model remains to be studied. Notably, eNOS‐derived NO promotes Notch signalling, cell proliferation, EMT and differentiation during heart development, and has been implicated to play a role in ROS handling.^35^, ^41^, ^42^ In the present study, although there was no difference in the total eNOS protein levels between all four groups, levels of phosphorylated eNOS varied, being the lowest in OMD and highest in the two groups with maternal exercise. Interestingly, Fukai et al^43^ demonstrated that exercise training increased eNOS and subsequently, extracellular SOD expression in vascular smooth muscle cells, leading to lower ROS levels. Our results indicate that eNOS function and oxidative stress in foetal hearts are improved with maternal exercise, which is conducive to proper cardiogenesis. However, whether the observed benefits of maternal exercise are eNOS and ROS dependent remains to be determined in future studies.

One limitation of this study is that the effects of exercise were not evaluated also using a model of type 2 diabetes, which is the more common type of diabetes in patients and could be examined in future studies. However, of note, large meta‐analyses have found that although women with type 2 diabetes achieve better glycemic control during pregnancy, the rate of malformations is not significantly different between type 1 and type 2 diabetes.^44^ There is evidence that glycemic status alone is not the most important factor in diabetic teratogenesis. Serum from diabetic animals with normalized glucose levels were found to be teratogenic in in vitro models.^45^ Also, numerous studies have shown that treatment with antioxidants alone are able to significantly reduce malformation rates in in vivo models, despite not having effects on hyperglycemic status.^13^, ^15^, ^34^, ^35^ While the present study did not show a direct causal relationship between reducing oxidative stress and beneficial effects of exercise, prior studies strongly indicate that oxidative stress is a key teratogenic component leading to diabetic embryopathy.^13^, ^15^, ^34^

To conclude, the results of our study indicate that maternal exercise protects against diabetes‐induced changes to embryonic gene expression, cell proliferation and EMT in embryonic hearts, possibly due to mitigation of oxidative stress. Future studies are required to provide more conclusive evidence to elucidate the molecular mechanisms by which maternal exercise benefits CHD pathogenesis. Studies in humans could be conducted to further evaluate the efficacy of maternal exercise in reducing CHD risk in pre‐gestational diabetes. Blood glucose levels were high in our animal model because diabetes was untreated, whereas better glycemic control is expected in pregnant women receiving pre‐natal care. Unfortunately, although the risks of birth defects due to hyperglycemia have become well known and modern prenatal care emphasizes optimal glucose control before conception, the incidence of CHDs in the offspring of diabetic women has not improved over time.^5^ Therefore, there is potential to further improve outcomes by modifying other teratogenic factors, in addition to maintaining glycemic control. Exercise may be a simple, yet effective intervention to supplement insulin therapy and minimize risks of birth defects in mothers with diabetes.

## CONFLICT OF INTEREST

The authors declare no conflict of interest.

## AUTHORS’ CONTRIBUTIONS

Conception and design of the research: TS, XL, DLJ, QF. Acquisition and analysis of data: TS, AE, ERG, MK, XL. Interpretation of the data: TS, AE, ERG, MK, XL, DLJ, QF. Statistical analysis: TS, QF. Drafting of the manuscript: TS. Supervision: DLJ, QF. Critical revision of the manuscript for important intellectual content TS, DLJ, QF. All authors read and approved the final manuscript.

## Data Availability

The data that support the findings of this study are available from the corresponding author upon reasonable request.

## References

[1] Hauser M . Congenital anomalies of the coronary arteries. Heart. 2005;91:1240‐1245. 16103577 10.1136/hrt.2004.057299 PMC1769093

[2] Gilboa SM , Devine OJ , Kucik JE , et al. Congenital heart defects in the United States: estimating the magnitude of the affected population in 2010. Circulation. 2016;134:101‐109. 27382105 10.1161/CIRCULATIONAHA.115.019307 PMC4942347

[3] Pierpont ME , Basson CT , Benson DW Jr , et al. Genetic basis for congenital heart defects: current knowledge: a scientific statement from the American Heart Association Congenital Cardiac Defects Committee, Council on Cardiovascular Disease in the Young: endorsed by the American Academy of Pediatrics. Circulation. 2007;115:3015‐3038. 17519398 10.1161/CIRCULATIONAHA.106.183056

[4] van der Bom T , Zomer AC , Zwinderman AH , Meijboom FJ , Bouma BJ , Mulder B . The changing epidemiology of congenital heart disease. Nat Rev Cardiol. 2011;8:50‐60. 21045784 10.1038/nrcardio.2010.166

[5] Øyen N , Diaz LJ , Leirgul E , et al. Prepregnancy diabetes and offspring risk of congenital heart disease: a nationwide cohort study. Circulation. 2016;133:2243‐2253. 27166384 10.1161/CIRCULATIONAHA.115.017465 PMC4890838

[6] Lisowski LA , Verheijen PM , Copel JA , et al. Congenital heart disease in pregnancies complicated by maternal diabetes mellitus. An international clinical collaboration, literature review, and meta‐analysis. Herz. 2010;35:19‐26. 20140785 10.1007/s00059-010-3244-3

[7] Wren C , Birrell G , Hawthorne G . Cardiovascular malformations in infants of diabetic mothers. Heart. 2003;89:1217‐1220. 12975424 10.1136/heart.89.10.1217 PMC1767924

[8] Marelli AJ , Ionescu‐Ittu R , Mackie AS , Guo L , Dendukuri N , Kaouache M . Lifetime prevalence of congenital heart disease in the general population from 2000 to 2010. Circulation. 2014;130:749‐756. 24944314 10.1161/CIRCULATIONAHA.113.008396

[9] Guariguata L , Whiting DR , Hambleton I , Beagley J , Linnenkamp U , Shaw JE . Global estimates of diabetes prevalence for 2013 and projections for 2035. Diabetes Res Clin Pract. 2014;103:137‐149. 24630390 10.1016/j.diabres.2013.11.002

[10] Opotowsky AR , Siddiqi OK , Webb GD . Trends in hospitalizations for adults with congenital heart disease in the U.S. J Am Coll Cardiol. 2009;54:460‐467. 19628123 10.1016/j.jacc.2009.04.037

[11] Schulkey CE , Regmi SD , Magnan RA , et al. The maternal‐age‐associated risk of congenital heart disease is modifiable. Nature. 2015;520:230‐233. 25830876 10.1038/nature14361 PMC4393370

[12] Brownlee M . Biochemistry and molecular cell biology of diabetic complications. Nature. 2001;414:813‐820. 11742414 10.1038/414813a

[13] Moazzen H , Lu X , Ma NL , et al. N‐Acetylcysteine prevents congenital heart defects induced by pregestational diabetes. Cardiovasc Diabetol. 2014;13:46. 24533448 10.1186/1475-2840-13-46 PMC3942143

[14] Matough FA , Budin SB , Hamid ZA , Alwahaibi N , Mohamed J . The role of oxidative stress and antioxidants in diabetic complications. Sultan Qaboos Univ Med J. 2012;12:5‐18. 22375253 10.12816/0003082 PMC3286717

[15] Moazzen H , Lu X , Liu M , Feng Q . Pregestational diabetes induces fetal coronary artery malformation via reactive oxygen species signaling. Diabetes. 2015;64:1431‐1443. 25422104 10.2337/db14-0190

[16] Moien‐Afshari F , Ghosh S , Khazaei M , Kieffer TJ , Brownsey RW , Laher I . Exercise restores endothelial function independently of weight loss or hyperglycaemic status in db/db mice. Diabetologia. 2008;51:1327‐1337. 18437348 10.1007/s00125-008-0996-x

[17] Engineer A , Saiyin T , Lu X , et al. Sapropterin treatment prevents congenital heart defects induced by pregestational diabetes in mice. J Am Heart Assoc. 2018;7:e009624. 30608180 10.1161/JAHA.118.009624 PMC6404194

[18] Liu Y , Lu X , Xiang F‐L , et al. Nitric oxide synthase‐3 deficiency results in hypoplastic coronary arteries and postnatal myocardial infarction. Eur Heart J. 2014;35:920‐931. 23048191 10.1093/eurheartj/ehs306

[19] Anderson RH , Spicer DE , Brown NA , Mohun TJ . The development of septation in the four‐chambered heart. Anat Rec (Hoboken). 2014;297:1414‐1429. 24863187 10.1002/ar.22949

[20] Lin C‐J , Lin C‐Y , Chen C‐H , Zhou B , Chang C‐P . Partitioning the heart: mechanisms of cardiac septation and valve development. Development. 2012;139:3277‐3299. 22912411 10.1242/dev.063495 PMC3424040

[21] Hans F , Dimitrov S . Histone H3 phosphorylation and cell division. Oncogene. 2001;20:3021‐3027. 11420717 10.1038/sj.onc.1204326

[22] Kumar SD , Dheen ST , Tay SS . Maternal diabetes induces congenital heart defects in mice by altering the expression of genes involved in cardiovascular development. Cardiovasc Diabetol. 2007;6:34. 17967198 10.1186/1475-2840-6-34 PMC2176054

[23] Schein PS , Loftus S . Streptozotocin: depression of mouse liver pyridine nucleotides. Cancer Res. 1968;28:1501‐1506. 4299824

[24] Hoffman JI , Kaplan S , Liberthson RR . Prevalence of congenital heart disease. Am Heart J. 2004;147:425‐439. 14999190 10.1016/j.ahj.2003.05.003

[25] Hornberger LK . Maternal diabetes and the fetal heart. Heart. 2006;92:1019‐1021. 16698822 10.1136/hrt.2005.083840 PMC1861084

[26] Liu S , Joseph KS , Lisonkova S , et al. Association between maternal chronic conditions and congenital heart defects: a population‐based cohort study. Circulation. 2013;128:583‐589. 23812182 10.1161/CIRCULATIONAHA.112.001054

[27] Zhou L , Liu J , Xiang M , et al. Gata4 potentiates second heart field proliferation and Hedgehog signaling for cardiac septation. Proc Natl Acad Sci USA. 2017;114:E1422‐E1431. 28167794 10.1073/pnas.1605137114 PMC5338429

[28] Oettgen P . Transcriptional regulation of vascular development. Circ Res. 2001;89:380‐388. 11532898 10.1161/hh1701.095958

[29] Tao J , Doughman Y , Yang KE , Ramirez‐Bergeron D , Watanabe M . Epicardial HIF signaling regulates vascular precursor cell invasion into the myocardium. Dev Biol. 2013;376:136‐149. 23384563 10.1016/j.ydbio.2013.01.026 PMC3602346

[30] Limbourg FP , Takeshita K , Radtke F , Bronson RT , Chin MT , Liao JK . Essential role of endothelial Notch1 in angiogenesis. Circulation. 2005;111:1826‐1832. 15809373 10.1161/01.CIR.0000160870.93058.DD PMC2633594

[31] von Gise A , Pu WT . Endocardial and epicardial epithelial to mesenchymal transitions in heart development and disease. Circ Res. 2012;110:1628‐1645. 22679138 10.1161/CIRCRESAHA.111.259960 PMC3427736

[32] Martínez‐Estrada OM , Lettice LA , Essafi A , et al. Wt1 is required for cardiovascular progenitor cell formation through transcriptional control of Snail and E‐cadherin. Nat Genet. 2010;42:89‐93. 20023660 10.1038/ng.494 PMC2799392

[33] von Gise A , Zhou B , Honor LB , et al. WT1 regulates epicardial epithelial to mesenchymal transition through beta‐catenin and retinoic acid signaling pathways. Dev Biol. 2011;356:421‐431. 21663736 10.1016/j.ydbio.2011.05.668 PMC3147112

[34] Sivan E , Lee Y‐C , Wu Y‐K , Reece EA . Free radical scavenging enzymes in fetal dysmorphogenesis among offspring of diabetic rats. Teratology. 1997;56:343‐349. 9485543 10.1002/(SICI)1096-9926(199712)56:6<343::AID-TERA1>3.0.CO;2-X

[35] Basu M , Garg V . Maternal hyperglycemia and fetal cardiac development: clinical impact and underlying mechanisms. Birth Defects Res. 2018;110:1504‐1516. 30576094 10.1002/bdr2.1435 PMC6310016

[36] Ayala A , Munoz MF , Arguelles S . Lipid peroxidation: production, metabolism, and signaling mechanisms of malondialdehyde and 4‐hydroxy‐2‐nonenal. Oxid Med Cell Longev. 2014;2014:360438. 24999379 10.1155/2014/360438 PMC4066722

[37] Radak Z , Zhao Z , Koltai E , Ohno H , Atalay M . Oxygen consumption and usage during physical exercise: the balance between oxidative stress and ROS‐dependent adaptive signaling. Antioxid Redox Signal. 2013;18:1208‐1246. 22978553 10.1089/ars.2011.4498 PMC3579386

[38] Fisher‐Wellman K , Bloomer RJ . Acute exercise and oxidative stress: a 30 year history. Dyn Med. 2009;8:5553. 10.1186/1476-5918-8-1 PMC2642810 19144121

[39] Gomes EC , Silva AN , de Oliveira MR . Oxidants, antioxidants, and the beneficial roles of exercise‐induced production of reactive species. Oxid Med Cell Longev. 2012;2012:756132. 22701757 10.1155/2012/756132 PMC3372226

[40] Chung E , Joiner HE , Skelton T , Looten KD , Manczak M , Reddy PH . Maternal exercise upregulates mitochondrial gene expression and increases enzyme activity of fetal mouse hearts. Physiol Rep. 2017;5:e13184. 28292876 10.14814/phy2.13184 PMC5350185

[41] Liu Y , Feng Q . NOing the heart: role of nitric oxide synthase‐3 in heart development. Differentiation. 2012;84:54‐61. 22579300 10.1016/j.diff.2012.04.004

[42] Basu M , Zhu J‐Y , LaHaye S , et al. Epigenetic mechanisms underlying maternal diabetes‐associated risk of congenital heart disease. JCI Insight. 2017;2 pii: 95085. 29046480 10.1172/jci.insight.95085 PMC5846898

[43] Fukai T , Siegfried MR , Ushio‐Fukai M , Cheng Y , Kojda G , Harrison DG . Regulation of the vascular extracellular superoxide dismutase by nitric oxide and exercise training. J Clin Invest. 2000;105:1631‐1639. 10841522 10.1172/JCI9551 PMC300857

[44] Balsells M , Garcia‐Patterson A , Gich I , et al. Maternal and fetal outcome in women with type 2 versus type 1 diabetes mellitus: a systematic review and metaanalysis. J Clin Endocrinol Metab. 2009;94:4284‐4291. 19808847 10.1210/jc.2009-1231

[45] Eriksson UJ , Wentzel P . The status of diabetic embryopathy. Ups J Med Sci. 2016;121:96‐112. 27117607 10.3109/03009734.2016.1165317 PMC4900070

